# Chitosan-Based Functional Films Integrated with Magnolol: Characterization, Antioxidant and Antimicrobial Activity and Pork Preservation

**DOI:** 10.3390/ijms22157769

**Published:** 2021-07-21

**Authors:** Xueying Song, Liu Liu, Xiaoxia Wu, Yongfeng Liu, Jialu Yuan

**Affiliations:** College of Food Engineering and Nutritional Science, Shanxi Normal University, Xi’an 710119, China; sxy18734445184@163.com (X.S.); xiaoxiaw@snnu.edu.cn (X.W.); yongfeng200@126.com (Y.L.); jialuuyuan@126.com (J.Y.)

**Keywords:** magnolol, chitosan, film, antimicrobial, pork

## Abstract

The aims of this study were to develop the magnolol–chitosan films and study the positive effect of the combination of magnolol and chitosan. The addition of magnolol made the magnolol–chitosan films exhibit higher density (1.06–1.87 g/cm^3^), but the relatively lower water vapor permeability (12.06–7.36 × 10^−11^·g·m^−1^·s^−1^·Pa^−1^) and water content (16.10–10.64%). The dense and smooth surface and cross-section of magnolol–chitosan films were observed by environmental scanning electron microscopy (ESEM) images. The interaction of magnolol and chitosan was observed by X-ray diffraction (XRD), Fourier transform infrared (FTIR) spectroscopy and thermogravimetric analysis (TGA). After the addition of magnolol, the antioxidant capacity of magnolol–chitosan films was increased from 18.99 to 82.00%, the growth of *P. aeruginosa* was inhibited and the inhibition percentage of biofilm formation was increased from 30.89 to 86.04%. We further verified that the application of magnolol–chitosan films on chilled pork significantly reduced the increases in pH value, inhibited the growth of microorganisms and extended the shelf life. Results suggest that magnolol had a positive effect on magnolol–chitosan films and could be effectively applied to pork preservation.

## 1. Introduction

Owing to the abundant nutrients of meat, it is a natural medium for spoilage micro-organisms and food-borne pathogens [1]. Fresh meat undergoes significant adverse quality changes during storage at 4 °C; the changes can be observed in pH value, flavor and so on [2]. Therefore, it is necessary that scientific treatments are used to delay the loss of freshness. During storage, microbial accumulation is an important reason for the deterioration of fresh meat. Inhibition of microbial spoilage and reduction of biofilm formation and microbial drug resistance are the primary strategies against microbe-induced spoilage in meat preservation [3].

*Pseudomonas aeruginosa* (*P. aeruginosa*) is a common food-borne pathogen, mainly found in meat and sewage [4]. During spoilage of meat, *P. aeruginosa* is dominant due to its fast growth rate and affinity for oxygen. Various types of food problems caused by *P. aeruginosa* further endanger people’s health worldwide [5]. In addition, *P. aeruginosa* can interact with other bacteria, fungi and viruses, which undoubtedly weakens the therapeutic effects of exogenous stresses [6]. Moreover, bacterial communities embedded in an extracellular matrix (ECM) form biofilms. *P. aeruginosa* biofilms are more resistant to host responses than their planktonic counterparts, which makes it difficult to remove the biofilms [7,8]. In order to solve the problem of food corruption caused by *P. aeruginosa*, it is necessary to continuously develop and improve effective antimicrobial strategies.

Active packaging, edible packaging, vacuum packaging, modified atmosphere packaging and intelligent packaging have been developed in meat preservation [3]. Currently, edible film or coating has advantages over synthetic materials in biodegradability and environmental protection, and composite active films combined with natural substances are developed as substitutes for traditional non-biodegradable plastic films [9,10]. Polysaccharides, including starch, cellulose and chitosan, have been used to develop food packages [11]. Chitosan-based coatings are considered the best edible and biologically safe preservative coatings for different foods, owing to their safety, biodegradability and antimicrobial activity [12]. Studies reported that edible coatings have the potential to improve food appearance and extend food shelf life [13,14]. Antibacterial food packaging is a new type of active packaging compound combined with natural antibacterial substances that reduce food spoilage [15]. Phenolic compounds are biologically active antioxidants and antibacterial agents that have been widely used in the food industry. Further research is needed on the preparation of bio-anticorrosive film by fusion of polyphenols and chitosan [16]. Previous studies have reported that the combination of phenolic compounds and chitosan film is often beneficial for various film properties. Magnolol is a bioactive polyphenolic component derived from the root and stem bark of *Magnolia officinalis*. Magnolol has been found to possess extensive biological properties, such as anti-inflammation, antioxidation and antimicrobial activities [17,18]. Magnolol shows significant antifungal activity against clinically isolated *Candida albicans* (*C. albicans*) strains in the concentration range of 16–32 μg/mL [19]. A previous study has also shown that magnolol can inhibit the secretion of α-toxin by *Staphylococcus aureus* (*S. aureus*) in subinhibitory concentrations [20]. However, there has been less research on the antibacterial activity and application of magnolol–chitosan films.

This study aimed to study the effect of magnolol incorporation on the antioxidant, antibacterial and antibiofilm properties of chitosan film. With fresh pork as a model, the application of magnolol–chitosan film was also evaluated.

## 2. Results and Discussion

### 2.1. Film Characterization

#### 2.1.1. Film Thickness and Density Analysis

**Figure 1:**
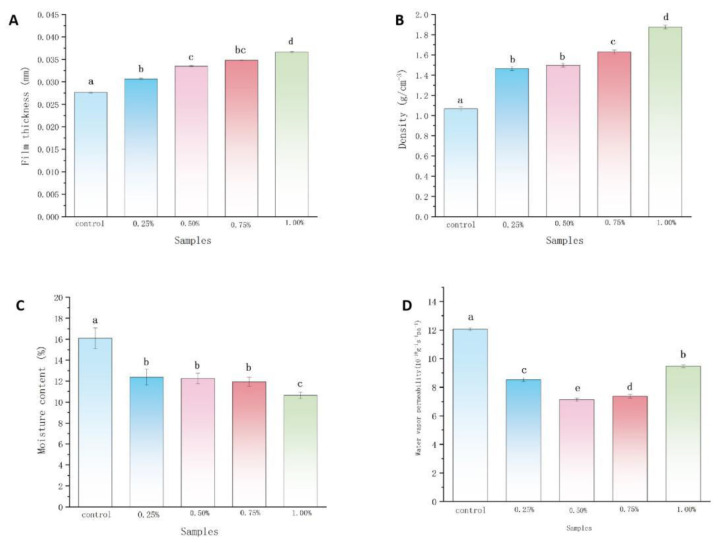
Thickness (**A**), density (**B**), moisture content (**C**) and water vapor permeability (WVP) (**D**) of chitosan films without and with different contents of magnolol. Notes: 0%, magnolol-chitosan film without magnolol. 0.25%, magnolol-chitosan film with 0.25% of magnolol. 0.50%, magnolol-chitosan film with 0.50% of magnolol. 0.75%, magnolol-chitosan film with 0.75% of magnolol. 1.00%, magnolol-chitosan film with 1.00% of magnolol.

The thicknesses and density of films are shown in Figure 1A,B. The changes in thicknesses and density of films are closely related to the addition of magnolol. With the increase in magnolol loading in the magnolol–chitosan film from 0 to 1%, the thickness and density of chitosan films significantly increased from 0.027 to 0.036 mm and 1.06–1.87 g/cm^3^, respectively. The increases in thickness and density of the film are in agreement with results found for tea polyphenol chitosan film [21]. The formation of high density may be due to the existence of polyhydroxyl groups, which are important bridges for binding multiple chitosan molecules. As the concentration of polyphenols increased, the effectiveness of hydrogen bonding increased, as did the hydrophobicity between polyphenols and chitosan [22]. The distance between chitosan molecules became shorter, and the film structure was more compact; thus, thickness and density were increased.

#### 2.1.2. Moisture Content and WVP

The moisture content is shown in Figure 1C. All the composite films had low moisture contents ranging from 10 to 20%. The highest moisture content was found in the control group, and the decreased moisture contents of the other groups were attributed to the increase in magnolol ratio. A similar decreased value was obtained in chitosan film incorporated with apple polyphenols [23]. When the polyphenolic compounds were added to the chitosan film, the hydroxyl and carboxyl of polyphenols interacted with chitosan. Due to the competitive binding effect, the interactions between hydrophilic groups of chitosan and water molecules were limited [24,25]. Therefore, less chitosan combined with water molecules, and the water content became lower. Film as a barrier to separate foods from the air can keep foods fresh. The addition of magnolol improved the water barrier property of the films, which was consistent with the trend of densification of the films. The permeation of water vapor at a given temperature was evaluated by WVP (Figure 1D). The addition of magnolol could weaken the WVP of the chitosan film. With the increase in magnolol concentration from 0 to 1.0%, the WVP of the films decreased from 12.06 to 7.36 × 10^−11^·g·m^−1^·s^−1^·Pa^−1^. Similar WVP results were observed in other chitosan–phenolic films, such as chitosan–gallic acid film and chitosan–protocatechuic acid film [26,27]. The decrease in the WVP was likely due to magnolol increasing the thickness and density of chitosan films. The magnolol–chitosan film prevents (or delays) the water vapor molecules from permeating [23].

#### 2.1.3. Color Analysis

**Figure 2:**
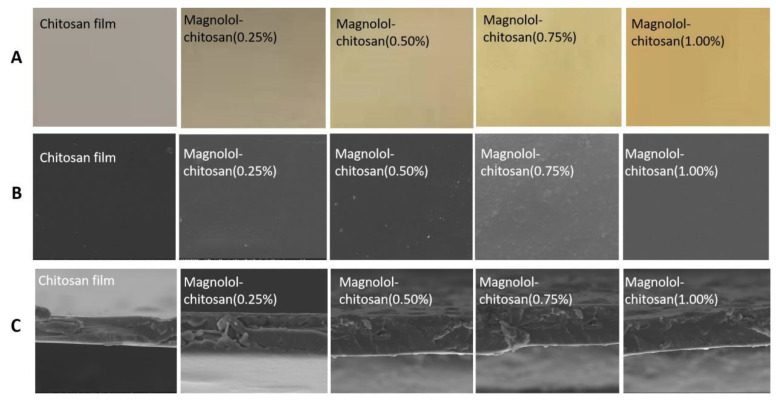
Photographs (**A**) and ESEM micrographs of surface (**B**) and cross-section (**C**) of chitosan films without and with different contents of magnolol.

The color parameters are summarized in Table 1. All the parameters were observed to be affected by magnolol addition. The control group had a light yellow appearance. After the addition of magnolol, the a* and b* values were significantly increased, while the L* value decreased, indicating a tendency towards yellowness and darkness. These color parameters are consistent with the physical appearances of chitosan film and magnolol–chitosan films (Figure 2A). Similar color changes were observed in films incorporated with different spice extracts [25]. Because the preservation of food is threatened by visible and ultraviolet light, the barrier of the protection of the food from the light is necessary. Generally speaking, a film close to yellowness could protect the packaged foods from exposure to light and slows lipid oxidation of foods, which is helpful in delaying the loss of original food quality [28]. In addition, magnolol–chitosan films could absorb ultraviolet and visible light radiation due to the aromatic groups [29].

#### 2.1.4. Microstructure of Magnolol–Chitosan Films

The ESEM images of the magnolol–chitosan films are shown in Figure 2B,C. The surface of the chitosan film was smooth without any macropores, which could be explained by a tight arrangement of polymer chains [25]. The addition of magnolol in chitosan film caused a rougher surface compared with chitosan film, but no surface break was observed. The appearance of the films was similar to that reported for chitosan incorporated into protocatechuic acid [27]. Our results indicate that the magnolol was uniformly distributed in the chitosan film. The cross-sections of magnolol–chitosan films showed more dense structures; the beneficial change of magnolol addition may be due to the interaction between the chitosan and the polyphenols and the presence of ordered crystalline regions [30]. Therefore, the addition of magnolol resulted in a denser structure.

#### 2.1.5. FTIR Spectroscopy Analysis

**Figure 3:**
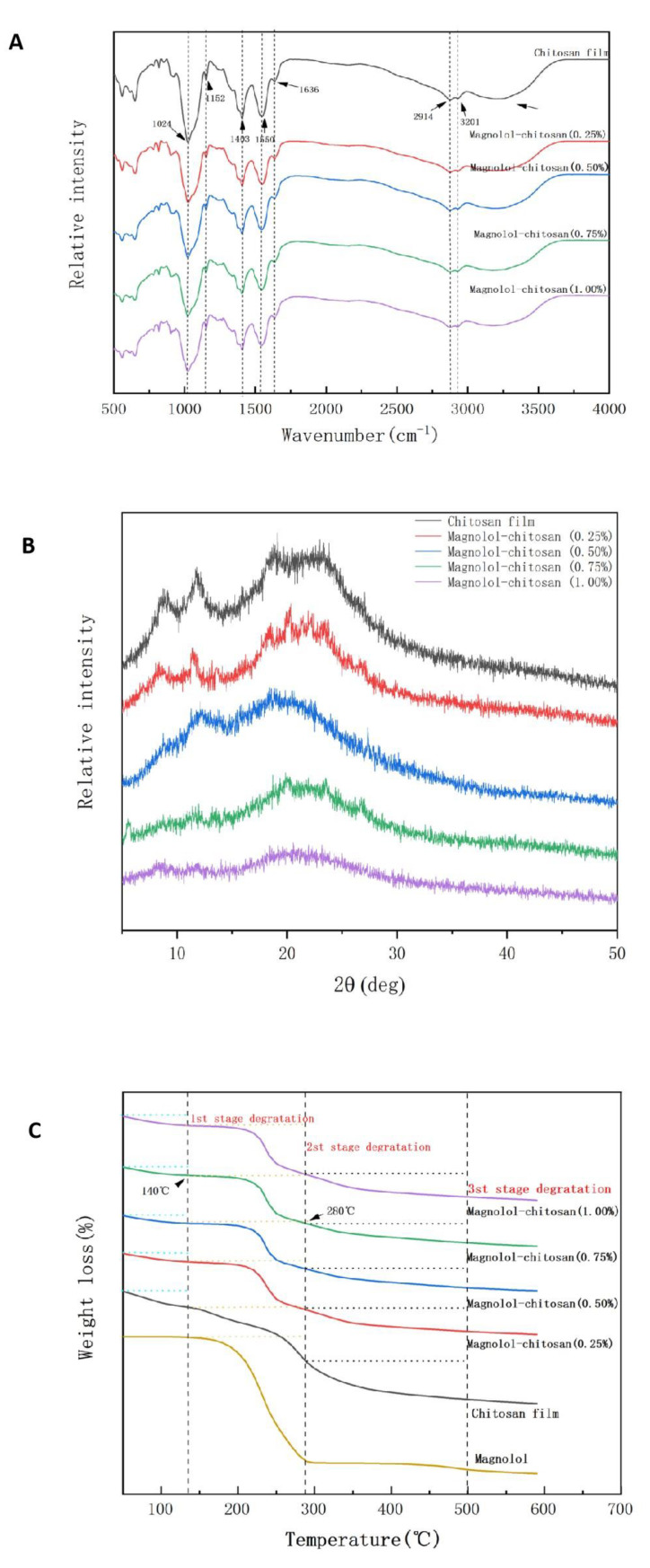
FTIR spectra (**A**), XRD patterns (**B**) and TGA (**C**) analysis of chitosan films without and with different contents of magnolol.

FTIR spectroscopy was used to analyze the functional groups of magnolol–chitosan films and explain the film structure constitution. As shown in Figure 3A, a broad absorption peak was found between 3500 and 3000 cm^−1^, attributed to the stretching vibration of O-H and N-H [15]. The bands at 2914 and 2877 cm^−1^ were representative of C-H vibrations. Besides, the peaks at 1636, 1550, 1403, 1152 cm^−1^ corresponded to C=O bending vibration, N-H bending vibration, C-N stretching and C-O-C band stretching, respectively [23]. Compared with the chitosan film, all the magnolol–chitosan film bands were roughly similar to the chitosan film bands; after adding magnolol, no additional peaks or obvious displacements were observed, indicating there was no chemical reaction between the two compounds [25]. The peak became more flattened with the magnolol added, due to the hydrogen bonding and hydrophobic force formed between polyphenols and chitosan [21]. It has been reported that the interaction between the OH group of polyphenols and the OH or NH group of chitosan leads to the interaction between the two compounds [21].

#### 2.1.6. XRD Analysis

X-ray diffraction was used to analyze the crystallization characteristics of magnolol–chitosan films. Noncrystalline, hydrated crystalline and anhydrous crystalline are three forms of chitosan. As revealed in Figure 3B, the results showed that the chitosan film had a crystalline structure. In the magnolol–chitosan film, there were four main diffraction peaks at 2θ = 8.57° (hydrated crystalline), 11.66° (anhydrous crystalline), 18.40° (hydrated crystalline) and 22.93° (noncrystalline), which were similar to the diffraction peaks of chitosan film incorporated with thinned young apple polyphenols [23]. The addition of magnolol limited the formation of hydrogen bonds between chitosan and magnolol, which formed a relatively amorphous complex with chitosan [31]. As the magnolol was incorporated into a chitosan film, the diffraction peak at 22.93° became lower and the crystalline peaks (8.57° and 11.66°) of chitosan films gradually disappeared. In the magnolol–chitosan films, the interactions between magnolol and chitosan may have limited the molecular hydrogen bond formation between chitosan molecules because of the competitive effect of hydrogen bonds between chitosan and magnolol, resulting in a low crystallinity. Thus, the amorphous structures in chitosan films appeared after the addition of magnolol [23].

#### 2.1.7. TGA Analysis

TGA is regarded as the most effective method for studying the thermal stability of films. Figure 3C displays the TGA curves of magnolol–chitosan films. Previous studies have shown that the thermal degradation of chitosan films occurs through a three-step weight loss [32]. The TGA curves of the chitosan film showed three decomposition stages of 50–140 °C, 140–280 °C and 280–500 °C. The first stage from 50 to 140 °C was due to the loss of free water. The second phase (140–280 °C) was explained as evaporation of bound water and glycerol. Further weight loss in the third stage was observed between 280 and 500 °C, which was due to the decomposition of polymers and breaking of the functional-group-associated chains in films [33]. Similar curves could be observed in magnolol–chitosan films. The thermal degradation of magnolol mainly occurred in the second stage. Compared with chitosan film, the main loss of magnolol–chitosan films did not change greatly as the concentration of magnolol increased, but the thermal degradation temperature of the magnolol–chitosan films decreased from 306.06 to 301.35 °C, which means that the thermal stability of the magnolol–chitosan films decreased. Generally, materials with higher crystallinity will have higher thermal stability, as more energy is required to fight a highly crystalline structure. Our results reveal that the processing temperature of magnolol–chitosan films reached 140 °C, which is suitable for use in food packaging.

### 2.2. Antioxidant Activity Analysis

**Figure 4:**
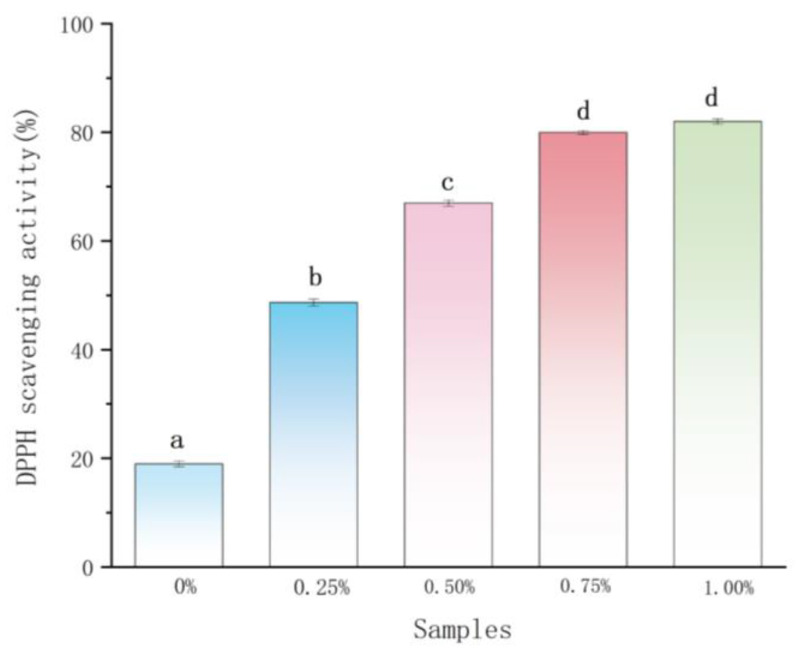
DPPH radical scavenging activity of chitosan films without and with different contents of magnolol. Notes: 0%, magnolol–chitosan film without magnolol. 0.25%, magnolol–chitosan film with 0.25% of magnolol. 0.50%, magnolol–chitosan film with 0.50% of magnolol. 0.75%, magnolol–chitosan film with 0.75% of magnolol. 1.00%, magnolol–chitosan film with 1.00% of magnolol.

Magnolol, which contains an allyl substituent in the structure, has high antioxidant activity [34]. Therefore, the effect of magnolol on the antioxidant activity of chitosan film was further explored. As outlined in Figure 4, the plain chitosan film showed the lowest antioxidant activity, the high antioxidant activity of magnolol–chitosan films was observed and the antioxidant activity presented elevation after the magnolol content was increased. This result indicated that the antioxidant activity of composite films mainly originated from the magnolol [24]. The chitosan matrix can protect magnolol during film processing and storage, while the excellent antioxidant activity of magnolol enhances the antioxidant activity of the film [30]. Liu et al. reported enhanced antioxidant activity upon adding spice extracts compared to plain chitosan film [25]. The excellent antioxidant ability of magnolol–chitosan films could be effective against food oxidation.

### 2.3. Antibacterial Activity on Planktonic P. aeruginosa

**Figure 5:**
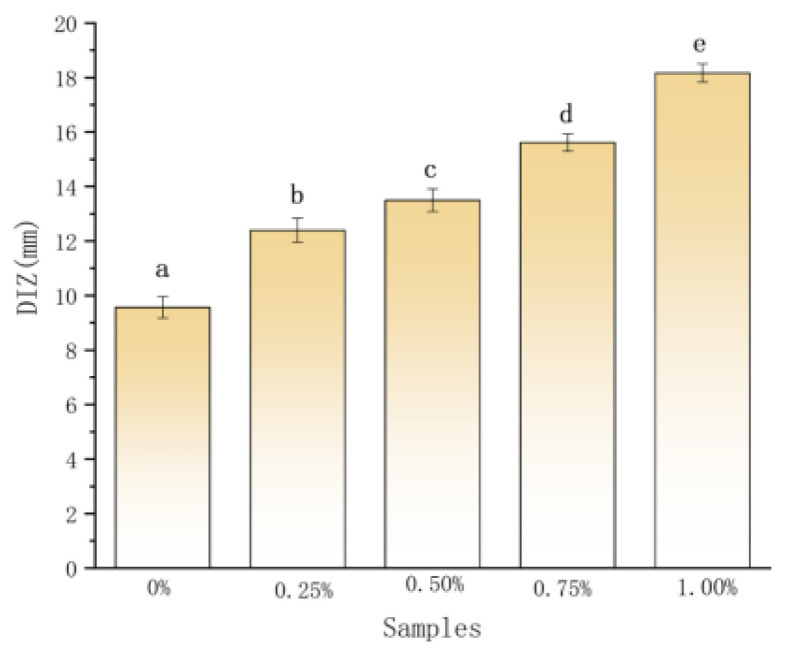
The diameter of inhibition zone (DIZ) of chitosan films without and with different contents of magnolol. Notes: 0%, magnolol–chitosan film without magnolol. 0.25%, magnolol–chitosan film with 0.25% of magnolol. 0.50%, magnolol–chitosan film with 0.50% of magnolol. 0.75%, magnolol–chitosan film with 0.75% of magnolol. 1.00%, magnolol–chitosan film with 1.00% of magnolol.

The antibacterial activity of the films is shown in Figure 5. The magnolol–chitosan films exhibited a patent inhibition zone for *P. aeruginosa*. As the concentration of magnolol increased, the diameter of the inhibitory zone (DIZ) was increased from 9.56 to 18.16 mm [15]. Similar antimicrobial activity was observed in the chitosan films incorporated with syringic acid [16]. The results suggested that the films prepared with magnolol presented improved antimicrobial activity compared to plain chitosan films, and the content of magnolol in the film was proportional to the antibacterial activity. Previous studies reported that magnolol and chitosan can inhibit the growth of various bacteria, such as *Staphylococcus aureus* (*S. aureus*) and oral pathogens [17,20]. The free amino groups of chitosan and phenolic compounds may act on the cell membrane of bacteria, causing loss of nutritional components of the cell and eventually resulting in bacteria death [25]. Therefore, the antimicrobial activities of magnolol–chitosan films may be the outcome of the functions of both magnolol and chitosan. The magnolol–chitosan film could be used as an antibacterial packaging material to extend the food shelf life.

### 2.4. Inhibition of Biofilm Formation

**Figure 6:**
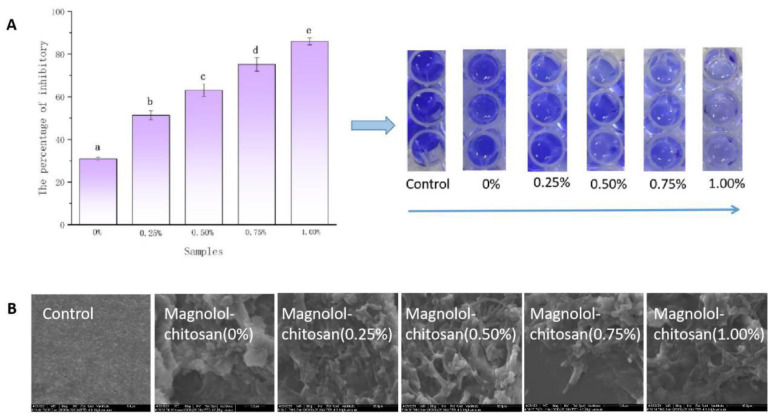
Antibiofilm activities of chitosan films without and with different contents of magnolol (**A**) and ESEM observation of biofilm formation (**B**). Notes: 0%, magnolol–chitosan film without magnolol. 0.25%, magnolol–chitosan film with 0.25% of magnolol. 0.50%, magnolol–chitosan film with 0.50% of magnolol. 0.75%, magnolol–chitosan film with 0.75% of magnolol. 1.00%, magnolol–chitosan film with 1.00% of magnolol.

Based on the ideal antibacterial activity of magnolol–chitosan films against *P. aeruginosa*, we further studied its antibiofilm activity. As seen in Figure 6A,B, when the *P. aeruginosa* biofilms were treated with magnolol–chitosan films, the biofilm quantities were significantly reduced; the highest inhibition percentage of biofilm was up to 86.04%. The good inhibitory effect on biofilm formation was the same as that of magnolol on the biofilm formation of *C. dubliniensis* [18]. Untreated *P. aeruginosa* biofilm displayed an intact and dense structure; after treatment with magnolol–chitosan films, the dense structure of biofilm disappeared and cells were damaged [35]. Summarizing the results, all films effectively inhibited *P. aeruginosa* biofilm formation, and the inhibition effect was dependent on the concentration of magnolol. Antibiofilm properties of magnolol against fungi and bacteria have been widely reported. Previous research has shown that lipophilic magnolol inhibits the biofilms formation dose-dependently through penetrating biofilm and targeting the complete cell wall and cell membrane structures [36,37]. Sun et al. revealed that magnolol (32 μg/mL) significantly inhibits the biofilm formation in the initial adhesion and the developmental phase [38]. The excellent antibiofilm properties of materials are profitable for food preservation to prevent the adhesion of microorganisms on the food.

### 2.5. Applications in Pork Preservation

**Figure 7:**
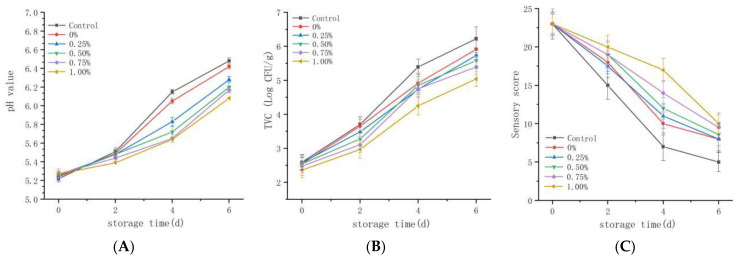
The pH values (**A**), TVC (**B**) and sensory evaluation (**C**) of pork samples during storage at 4 °C for 6 days. Notes: 0%, magnolol–chitosan film without magnolol. 0.25%, magnolol–chitosan film with 0.25% of magnolol. 0.50%, magnolol–chitosan film with 0.50% of magnolol. 0.75%, magnolol–chitosan film with 0.75% of magnolol. 1.00%, magnolol–chitosan film with 1.00% of magnolol.

Based on the good antioxidant and antibacterial properties of the magnolol–chitosan film, it was applied in pork preservation. The changes in pH value for all samples are given in Figure 7A. Regarding pH value, the initial pH values of pork samples were between 5 and 5.5, and the pH values kept rising during storage at 4 °C. Noticeably, the pH values of treated pork were effectively controlled. At the end of storage, the pork loaded with chitosan in combination with 1% magnolol had the lowest pH value [25,38]. Proteins in pork samples were degraded into volatile alkaline nitrogen molecules through microbial activity and meat endogenous proteases, resulting in an increase in pork pH. The more stable pH value of pork was due to the good antioxidant activity and antibacterial activity of magnolol–chitosan films, which inhibited protein degradation and produced fewer amines [39].

To validate the inhibitory effect of magnolol–chitosan film on the growth of microorganisms in pork samples, the TVC was measured and is shown in Figure 7B. The results indicated that the TVC of initial pork samples was low and gradually increased during storage at 4 °C. It was worth noting the TVC of the control group exceeded 6 log CFU/g on day 6, while magnolol–chitosan film loading induced a decrease in TVC, and all the treated samples still had a TVC less of than 6 log CFU/g after 6 days of storage [25]. With regard to pork safety, untreated pork was beyond acceptable limits, and magnolol–chitosan films effectively prolonged the shelf life of pork by controlling the proliferation of bacteria. The TVC changes of uncoated and coated pork samples were similar to the effect of chitosan/nisin/gallic acid coating on the preservation of pork loin [40]. Chitosan coating has previously been reported to have an inhibitory effect on the growth of yeasts, molds and Gram-positive and Gram-negative bacteria [41]. This might be attributed to the good barrier property and antibiotic activity of the edible magnolol–chitosan coating.

Sensory evaluation is an important index of meat quality. Based on the color, texture, odor, viscidity and broth, the pork samples were scored on days 0, 2, 4 and 6; the total scores are depicted in Figure 7C. The pork quality as determined by sensory evaluation was consistent with the above experimental analysis. With or without coating, the initial qualities of pork were generally satisfactory, and the quality of pork continued to decline during the 6 days storage. However, the coating of magnolol–chitosan films could better maintain the quality of pork. The control group showed slight odor and viscosity on day 2, while the pork coated with magnolol–chitosan films produced odor on day 4. After 6 days of storage, the control group had an unacceptable putrid odor and the color became yellow, while the coating groups were generally given acceptable evaluation. In a similar study, Ruan et al. also showed that the sodium alginate and carboxymethyl cellulose edible coating with epigallocatechin gallate active coating solution had no adverse effects on the sensory attributes of pork samples and improved the overall acceptance of pork samples [41]. During pork storage, microbial inhibition and lipid oxidation inhibition of magnolol–chitosan membrane during pork storage are the important reasons for good sensory quality.

In summary, magnolol–chitosan films could effectively prolong the preservation of pork, and the increased magnolol concentration is beneficial for pork preservation.

## 3. Materials and Methods

### 3.1. Materials and Reagents

Chitosan (degree of deacetylation 90%, viscosity 200–400 mPa·s) and Magnolol were purchased from the Aladdin Chemical Co. (Shanghai, China). All chemicals (analytical grade) were bought from Jingbo Chemical Co. (Xi’an, China). *P. aeruginosa* ATCC 10145 strain was obtained from the Food Safety and Health Laboratory, Food Engineering and Nutrition Science College (Shaanxi Normal University, Xi’an, China). The *P. aeruginosa* strains were shake cultured at 37 °C for 16 h and diluted with Luria–Bertani (LB) broth to the OD_600nm_ value of approximately 0.5.

### 3.2. Preparation of Chitosan Films

The compositions of five chitosan films are shown in Table 2. The chitosan solutions were prepared by dissolving chitosan with 1% acetic acid solution. Then, glycerol was added and stirred as a plasticizer; after 60 min agitation, different amounts of magnolol were stirred into the chitosan solution, and the bubble of the film-forming solution was degassed. Ten milliliters of chitosan active solution was poured into a circular container and kept for 48 h at room temperature. After film formation, the films were stored in a desiccator (relative humidity 53%) containing dried Mg(NO_3_)_2_ for at least 48 h before further analysis.

### 3.3. Application of Fresh Pork

The fresh pork was purchased and transferred in a low-temperature environment. The pork was processed using sterile tools and randomly divided into six groups. The control group was directly stored at 4 °C; test groups were immersed in different magnolol–chitosan solutions for 30 s before stored. The pH value, total viable count (TVC) and sensory evaluation measurements were performed on days 0, 2, 4, and 6 according to Miao et al. [39].

### 3.4. Characterization Methods

#### 3.4.1. Film Thickness and Density Assays

The film thickness was evaluated using a micrometer (Japan SANXING Co., Shandong, China). Six different locations on the films were selected and measured; the mean was used as the thickness of the film.

The density of films was calculated by the following equation:

(1) $$\mathrm{Density}=\frac{\mathrm{weight}\;(g)}{{\mathrm{area}\;(\mathrm{cm}}^{2})\times \mathrm{thickness}\;(\mathrm{cm})}$$

#### 3.4.2. Color Assay

The color parameters of each film were measured by using a Konica colorimeter (CR-300, Ichihara, Japan). Color values of L* (lightness), a* (green–red) and b* (blue–yellow) were measured to characterize each film.

The total color difference (ΔE) and whiteness index (WI) of films were calculated as follows:

(2) $$\Delta E=\sqrt{{\left(a*-a\right)}^{2}+{\left(b*-b\right)}^{2}+{\left(c*-c\right)}^{2}}$$

(3) $$\mathrm{WI}=100-\sqrt{{(100-L)}^{2}+a^{2}+b^{2}}$$

The color parameters of standard white plate were L*, a* and b* (L* = 98.09, a* = 0.40 and b* = 1.02), and the color parameters of the film sample were L, a, and b.

#### 3.4.3. Moisture Content and Water Solubility Assays

The film was weighed as an initial weight (M_1_) and then weighed again after it was dried to a constant weight at 105 °C (M_2_). In the water solubility study, the dried film was placed in a beaker containing distilled water, and the beaker was sealed with plastic wrap for 24 h. Then, the film was removed, rapidly dried, and weighed again after being dried at 105 °C to a constant weight (M_3_).

The moisture content and water solubility of the film were calculated as follows:

(4) $$\mathrm{Moisture}\;\mathrm{content}\;(\%)=\frac{M_{1}\;-\;M_{2}}{M_{1}}\times 100\%$$

(5) $$\mathrm{Water}\;\mathrm{solubility}\;(\%)=\frac{M_{2}\;-\;M_{3}}{M_{2}}\times 100\%$$

#### 3.4.4. WVP Assay

Each film was fixed in a glass cup with anhydrous calcium chloride inside. After being weighed, the cup was placed in a desiccator and weighed daily until the mass change was <0.001 g.

The water vapor permeability (WVP) of the film was calculated as follows:

(6) $$\mathrm{Water}\;\mathrm{vapor}\;\mathrm{permeability}\;=\frac{W\;\times \;X}{t\;\times \;A\;\times \;\Delta P}$$

The W(g) represents the increased weight, X(m) represents the thickness, t(s) represented the time required to reach a constant weight, A(m^2^) represents the infiltration area, and P is the partial vapor pressure difference between the dry atmosphere and the pure water (2339 Pa at 20 °C).

### 3.5. Structural Characterization of Films

#### 3.5.1. ESEM Assay

The cross-sectional and surface morphology of the film’s microstructure was examined by ESEM (FEI-Quanta 200, Hillsboro, OR, USA). The films were cut, fixed, coated on gold and observed.

#### 3.5.2. FTIR Spectroscopy Assay

Compatibility of film components was estimated through FTIR spectroscopy and recorded using a Nicolet iS10 spectrometer (Thermo Fisher Scientific Inc., Waltham, MA, USA). The wavenumber ranged from 4000 to 400 cm^−1^ at a resolution of 4 cm^−1^ and 16 scans.

#### 3.5.3. XRD Assay

The X-ray analyses of films were conducted using a diffractometer (D8 Advance, Brock, Germany). The scanning speed used was 5/min; 2θ ranged from 5 to 50°.

#### 3.5.4. Thermal Property Assay

Thermal properties of the films were measured by thermogravimetric analysis (TGA) with a Thermoanalyzer Systems instrument (Q1000DSC + LNCS + FACSQ600SDT, TA Instruments, New Castle, DE, USA). Samples were heated from 50 to 600 °C; the heating rate was 10 °C/min, and the nitrogen flow was 60 mL/min.

### 3.6. Antioxidant Activity Assay

The antioxidant activity was determined according to the method reported by Liu et al. and slightly modified [25]. The films were dissolved in acetic acid solution (1%). The diluted solution was added to the methanol solution of DPPH (0.1 mM) and incubated in a dark environment for 30 min. The absorbances of methanol solution of DPPH and samples were determined at 517 nm by a UV–visible spectrophotometer (UV 2100, Unico Instruments Co. Ltd., Shanghai, China) and marked as A_1_ and A_2_, respectively. DPPH radical scavenging activity was calculated by the following equation:

(7) $$\mathrm{DPPH}\;\mathrm{scavenging}\;\mathrm{activity}(\%)=\frac{A_{1}-A_{2}}{A_{1}}$$

### 3.7. Antibacterial Activity Assay

Antibacterial activity of film was tested according to the method of Kang et al. with some modifications [35]. One hundred microliters of *P. aeruginosa* suspension (OD_600nm_ = 0.5) was coated on the Mueller–Hinton agar (MHA) plates, which were prepared in glass Petri dishes. Oxford cups were placed on the plate, and 200 μL film solution was injected into the Oxford cup. The DIZ of magnolol–chitosan on *P. aeruginosa* was measured after incubation at 37 °C for 24 h.

### 3.8. Antibiofilm Assay

#### 3.8.1. Inhibitory Activities on Biofilm Formation

The protocol was adopted from Kang et al. [35], with slight modifications. The bacteria biofilm was formed in a 96-well polystyrene plate (JET Biofil, Guangzhou, China). The magnolol–chitosan was added at the beginning of the biofilm formation. *P. aeruginosa* suspension (OD_600nm_ = 0.5) in 1/8 LB broth with magnolol–chitosan was cultivated for 24 h at 37 °C. After incubation, PBS was used to wash the wells to remove the bacteria. The biofilms were fixed using methanol; 1% crystal violet staining solution was used as a stain. Ethanol (95%) was added to the 96-well plate, and the plate was kept at 37 °C for 20 min. The OD_590nm_ was measured using a Multiskan Spectrum spectrophotometer (Multiskan Go, Thermo Fisher Scientific, Waltham, MA, USA).

The inhibition percentage of biofilm formation was calculated as follows:

$$\mathrm{Inhibition}\;\mathrm{percentage}\;(\%)\;=(1\;-\;\frac{{\mathrm{OD}}_{590}\;(\mathrm{sample})}{{\mathrm{OD}}_{590}\;(\mathrm{crystal}\;\mathrm{violet}\;\mathrm{solution})})\;\times 100\%$$

#### 3.8.2. ESEM Analysis of *P. aeruginosa* Biofilm

The morphology of the *P. aeruginosa* biofilms was observed and recorded by using an ESEM (Quanta 200; FEI, Hillsboro, OR, USA). *P. aeruginosa* biofilms were formed on glass coverslips according to the method of Kang et al. [35]. The samples were fixed in 2.5% glutaraldehyde at 6 h. Afterward, the samples were dehydrated through an ethanol series. Finally, the biofilms were coated with gold and examined by ESEM.

### 3.9. Statistical Analysis

**Figure 8:**
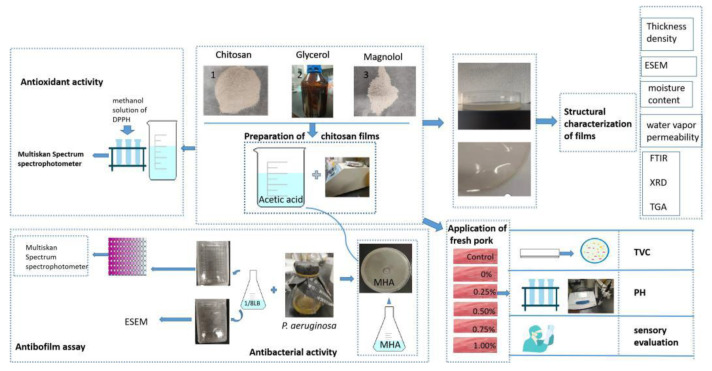
Method summary diagram.

All analyses were performed in triplicate and obtained data were expressed as mean and standard deviations (SD). The statistical analyses of the data were conducted using the IBM SPSS statistical software (version 21.0, IBM, Chicago, IL, USA), and least significant differences (*p* < 0.05) were accepted among the treatments (Figure 8).

## 4. Conclusions

In conclusion, natural magnolol was loaded in chitosan film with different ratios (0%, 0.25%, 0.5%, 0.75%, 1%), and the physical properties, antioxidant properties, antibacterial properties and application of magnolol–chitosan films were explored. The magnolol–chitosan films displayed higher thickness and density and lower water content and WVP relative to chitosan film. The good compatibility of magnolol–chitosan films was evidenced by ESEM and TGA. In addition, the incorporation of magnolol reduced the crystallinity of the chitosan film; the probable interaction between magnolol and chitosan was hydrogen bonding, which was confirmed by FTIR and XRD. Moreover, the magnolol–chitosan films displayed good antioxidant properties, antibacterial activity and antibiofilm properties. Finally, magnolol–chitosan films were used as a coating material in pork preservation. After coating, the increase in pH value and TVC and the decrease in sensory quality of pork were delayed, indicating that the magnolol–chitosan film has the potential to extend the shelf life of pork.

## Figures and Tables

**Table 1:** The color parameters of magnolol–chitosan films.

Color Parameters	Films				
	Magnolol–Chitosan (0%)	Magnolol–Chitosan (0.25%)	Magnolol–Chitosan (0.50%)	Magnolol–Chitosan (0.75%)	Magnolol–Chitosan (1.00%)
a*	−0.18 ± 0.01 ^d^	−0.12 ± 0.01 ^c^	0.11 ± 0.03 ^b^	0.25 ± 0.04 ^a^	0.28 ± 0.01 ^a^
b*	1.99 ± 0.05 ^e^	3.73 ± 0.04 ^d^	9.71 ± 0.01 ^c^	12.19 ± 0.06 ^b^	13.09 ± 0.02 ^a^
L*	67.40 ± 0.02 ^d^	62.22 ± 0.03 ^d^	60.11 ± 0.01 ^c^	57.21 ± 0.04 ^b^	51.79 ± 0.04 ^a^
ΔE	30.65 ± 0.02 ^e^	35.90 ± 0.02 ^d^	38.96 ± 0.01 ^c^	42.34 ± 0.06 ^b^	47.84 ± 0.01 ^a^
WI	67.39 ± 0.02 ^e^	62.02 ± 0.02 ^d^	58.94 ± 0.01 ^c^	55.54 ± 0.01 ^b^	50.04 ± 0.01 ^a^

Notes: Values are given as mean ± standard deviation (SD) of three independent tests. Different letters in the same row indicate significant difference (*p* < 0.05). The color parameters of standard white plate were L*, a* and b* (L* = 98.09, a* = 0.40 and b* = 1.02), and the color parameters of film sample were L, a, and b.

**Table 2:** The compositions of magnolol–chitosan films.

Sample Code	Magnolol Content				
	0%	0.25%	0.5%	0.75%	1%
Chitosan (g)	1 g	1 g	1 g	1 g	1 g
1% Acetic acid (mL)	50 mL	50 mL	50 mL	50 mL	50 mL
Glycerol (mL)	0.3 mL	0.3 mL	0.3 mL	0.3 mL	0.3 mL
Magnolol (g)	0 g	0.25 g	0.5 g	0.75 g	1 g

## Data Availability

Not applicable.
